# Sexualisierte Gewalterfahrungen, Bystander-Perspektiven und Disclosure junger Menschen – Ergebnisse aus der 10. Welle der Jugendsexualitätsstudie

**DOI:** 10.1007/s00103-026-04212-y

**Published:** 2026-03-27

**Authors:** Luise Dinger, Alina Schäfer-Pels, Sara Scharmanski

**Affiliations:** Abteilung S – Sexualaufklärung, Verhütung und Familienplanung, Referat S3 – Aufgabenkoordinierung, Nationale und internationale Zusammenarbeit, Forschung und Fortbildung, Bundesinstitut für Öffentliche Gesundheit (BIÖG), Maarweg 149–161, 50825 Köln, Deutschland

**Keywords:** Sexuelle Bildung, Prävention, Peer-Gewalt, Offenlegung, Adoleszenz, Jugend, Sexual education, Prevention, Peer violence, Disclosure, Youth, Prevalence

## Abstract

**Hintergrund:**

Das Bundesinstitut für Öffentliche Gesundheit (BIÖG, vormals Bundeszentrale für gesundheitliche Aufklärung, BZgA) erhebt seit 1998 im Rahmen der repräsentativen Wiederholungsstudie „Jugendsexualität“ Daten zu sexualisierten Gewalterfahrungen junger Menschen. Das Messinstrument wurde seither kontinuierlich angepasst. Auf Basis erster deskriptiver Befunde werden zentrale Ergebnisse der 10. Trendwelle vorgestellt. Diese umfassen die Prävalenzen körperlicher und nichtkörperlicher sexualisierter Gewalt sowie Daten zu den ausübenden Personen, zur Anwesenheit von Bystandern und dem Disclosure-Prozess.

**Methoden:**

Die Datenerhebung erfolgte im ersten Halbjahr 2025 mittels CAPI (Computer-assisted Personal Interviewing) in mündlich-schriftlichen Interviews mit Jugendlichen (14–17 Jahre) und jungen Erwachsenen (18–25 Jahre) (*N* = 5855). Der Fragenkatalog wurde u. a. um Items zu Bystandern und dem Disclosure-Prozess ergänzt.

**Ergebnisse:**

Die Ergebnisse belegen eine hohe Betroffenheit von körperlicher und nichtkörperlicher Gewalt im Jugendalter. Eine zentrale Rolle spielen Gleichaltrige sowohl bei der Ausübung sexualisierter Gewalt als auch als Bystander währenddessen und im Disclosure-Prozess.

**Diskussion:**

Die Studie bestätigt nationale und internationale Befunde, wonach das Jugendalter eine Phase erhöhten Risikos für sexualisierte (Peer‑)Gewalt ist, und unterstreicht die Bedeutung von Bystandern während und nach Situationen sexualisierter Gewalt. Interventions- und Präventionsmaßnahmen sollten daher gezielt auch Bystander adressieren.

**Zusatzmaterial online:**

Zusätzliche Informationen sind in der Online-Version dieses Artikels (10.1007/s00103-026-04212-y) enthalten.

## Abstract (en)

**Background:**

Since 1998, the Federal Institute for Public Health (BIÖG, formerly Federal Centre for Health Education, BZgA) has gathered data on young people’s experiences of sexual violence through the representative “Youth Sexuality” survey. The survey has been regularly updated and refined. Drawing on initial descriptive findings, the key results of the 10th wave are presented. These cover the prevalence of direct (hands-on) and indirect (hands-off) experience of sexual violence, information about perpetrators, the role of bystanders, and the process of disclosure.

**Methods:**

In the first half of 2025, computer-assisted personal interviews (CAPI) were conducted with a sample (*N* = 5855) of adolescents (14–17 years) and young adults (18–25 years). The latest wave of the representative survey included questions on bystanders and the disclosure process.

**Results:**

The results reveal a high prevalence of both direct and indirect sexual violence during adolescence. Peers play a key role—as perpetrators, as bystanders, and in the disclosure process.

**Discussion:**

The study supports national and international evidence that adolescence is a period of heightened risk for sexual (peer) violence. It highlights the crucial role of bystanders—both during and after sexual violence—suggesting that intervention and prevention efforts should also target bystanders.

## Hintergrund

Junge Menschen sind nachweislich einem erhöhten Risiko ausgesetzt, sexualisierte Gewalt zu erleben [1–11]. Wie hoch das Ausmaß in Deutschland ist, hat jüngst die bundesweite repräsentative Dunkelfeldstudie von Dreßling et al. (2025) erneut bestätigt, wonach 12,7 % aller Befragten zwischen 18 und 59 Jahren sexualisierte Gewalt im Kindes- oder Jugendalter erfahren haben [2].

Sexualisierte Gewalthandlungen umfassen körperliche oder nichtkörperliche Handlungen mit sexuellem Bezug ohne Einwilligung bzw. Einwilligungsfähigkeit der betroffenen Personen. Anders als in der Kindheit gehen diese Handlungen in der Adoleszenzphase häufig von Gleichaltrigen aus (Peer-Gewalt) – und dies nicht selten in Anwesenheit oder mit Kenntnis anderer Peers [1, 2, 4, 6–10, 12–24]. Auch der Disclosure-Prozess, also die prozesshafte Offenlegung sexualisierter Gewalterfahrungen, erfolgt in der Regel unter Jugendlichen ohne den Einbezug Erwachsener [4, 6, 8, 9, 16, 24–26]. Jugendliche können demnach Betroffene, Ausübende, aber auch Bystander sein. Als Bystander werden nach Doll et al. (2021) Personen verstanden, die als sogenannte Dritte während Situationen sexualisierter Gewalt (mit) anwesend sind oder danach von solchen erfahren [15].

Vor diesem Hintergrund hat das Bundesinstitut für Öffentliche Gesundheit (BIÖG)^1^ den Fokus der Befragung zu sexualisierter Gewalt in der Erfahrung junger Menschen geschärft [27]. Die Befragung ist eingebettet in die repräsentative Wiederholungsbefragung zum Sexual- und Verhütungsverhalten junger Menschen in Deutschland, die das BIÖG im Rahmen seines gesetzlichen Auftrags (Schwangerschaftskonfliktgesetz, § 1 SchKG) seit 1980 regelmäßig durchführt [4, 27–29].^2^

Im vorliegenden Beitrag werden erste deskriptive Ergebnisse der 10. Trendwelle vorgestellt. Im Fokus stehen die Prävalenzen (nicht-)körperlicher sexualisierter Gewalt in der Erfahrung Jugendlicher und junger Erwachsener sowie Angaben zu den ausübenden Personen bei der ersten körperlichen Gewalterfahrung. Umfassend berücksichtigt wird außerdem die Perspektive auf bzw. von Bystandern.

## Methoden

### Vorgehen

Die Befragung der jungen Menschen wurde von der Ethikkommission der Deutschen Gesellschaft für Psychologie (DGPs) umfassend geprüft (Ethikvotum vom 05.11.2024). Die Jugendlichen, ihre Erziehungsberechtigten sowie die jungen Erwachsenen wurden vorab mündlich und schriftlich über Ziel, Ablauf und datenschutzrechtliche Aspekte der Studie informiert. Voraussetzung für die freiwillige Teilnahme war ihre schriftliche Einwilligung sowie bei Minderjährigen die Zustimmung einer erziehungsberechtigten Person. Durch Schulungen der Interviewenden im Vorfeld sowie Supervisionsmöglichkeiten währenddessen konnte eine sensible und wertschätzende Befragung der jungen Menschen sichergestellt werden. Die Befragten wurden im Anschluss an das Interview auf Hilfs- und Beratungsangebote u. a. aus dem Bereich Prävention sexualisierter Gewalt hingewiesen. Die Datenerhebung erfolgte im ersten Halbjahr 2025. Weitere Details zum methodischen Vorgehen sind im Beitrag von Scharmanski und Dinger in diesem Themenheft zu finden.

### Stichprobe

Die Stichprobe besteht aus 5855 Personen: *n* = 3514 14- bis 17-jährige Jugendliche (davon Mädchen: *n* = 2013, Jungen: *n* = 1489, Personen ohne Geschlechtszuordnung: *n* = 12) und *n* = 2341 18- bis 25-jährige junge Erwachsene (darunter junge Frauen: 1543, junge Männer: 767, Personen ohne Geschlechtszuordnung: *n* = 31). Unter den Jugendlichen geben *n* = 3295 (94 %) an, mindestens vorwiegend heterosexuell orientiert zu sein, *n* = 147 (4 %) berichten von einer mindestens vorwiegend homosexuellen oder bisexuellen Orientierung. Weitere *n* = 72 (2 %) geben eine andere sexuelle Orientierung (z. B. pansexuell oder noch unsicher) an. Bei den jungen Erwachsenen geben *n* = 2018 (86 %) eine mindestens vorwiegend heterosexuelle Orientierung an, *n* = 271 (12 %) berichten von einer mindestens vorwiegend homosexuellen oder bisexuellen Orientierung, während *n* = 52 (2 %) eine andere sexuelle Orientierung angeben. Insgesamt haben *n* = 715 (20 %) der Jugendlichen bereits hetero- und/oder homosexuelle Erfahrungen gemacht, bei den jungen Erwachsenen sind es *n* = 1767 (75 %).

### Erhebungsinstrument

Neben demografischen Daten (z. B. Alter, Geschlecht und sexuelle Orientierung) wurden Angaben zu Erfahrungen sexualisierter Gewalt, den ausübenden Personen, der Bystander-Perspektive und dem Disclosure-Prozess erfasst.^3^

Erfahrungen sexualisierter Gewalt wurden operationalisiert durch Gewalterfahrungen *ohne* Körperkontakt (z. B. exhibitionistische Handlungen) sowie *mit* Körperkontakt (z. B. Berührungen gegen den eigenen Willen) und in Anlehnung an ein etabliertes Instrument [8] erfragt. Des Weiteren wurde die Prävalenz sexualisierter Gewalt unter Einbezug digitaler Medien erfragt (z. B. der Erhalt, die Aufnahme oder das Teilen von intimem Bild- und Videomaterial gegen den Willen der Befragten). Die Befragten, die angaben, mindestens eine Form sexualisierter Gewalt *mit *Körperkontakt erlebt zu haben, wurden außerdem zu ihrem Alter zum Tatzeitpunkt sowie detaillierter zu den ausübenden Personen befragt (Anzahl, Alter und Beziehung zur ausübenden Person). Sofern sie mehrere Formen erlebt hatten, sollten sie die erste Erfahrung als Referenz heranziehen.

Die Bystander-Perspektive wurde zum einen dadurch erhoben, dass alle Jugendlichen und jungen Erwachsenen gefragt wurden, inwiefern in ihrem Beisein oder mit ihrer Kenntnis eine andere Person zu sexuellen Handlungen gedrängt oder gezwungen wurde bzw. ob sie im Nachhinein davon erfahren haben. Zum anderen wurden Befragte in Bezug auf ihre erste sexualisierte Gewalterfahrung *mit* Körperkontakt um Angaben zur Anwesenheit von Bystandern während der Tat sowie zum Disclosure-Prozess gebeten (Zeitpunkt der Offenlegung, adressierte Personen, wahrgenommener Nutzen der Offenlegung sowie Gründe für die Nichtoffenlegung).

### Statistische Analysen

Im Vorfeld der statistischen Auswertung wurde der Datensatz bereinigt und aufbereitet. Während dieses Prozesses wurde die Geschlechterverteilung in der Stichprobe gewichtet, sodass die Ergebnisse einer repräsentativen Grundgesamtheit entsprechen. Grundlage hierfür waren Daten des Statistischen Bundesamts, die auf Anfrage zur Verfügung gestellt wurden ([30]; eine detaillierte Darstellung ist im Beitrag von Scharmanski und Dinger in diesem Themenheft zu finden). Die statistische Auswertung erfolgt durch deskriptive Analysen und mithilfe der Software IBM SPSS Version 25 (IBM, Armonk, NY, USA).

## Ergebnisse

### Häufigkeit sexualisierter Gewalterfahrungen

#### Sexualisierte Gewalterfahrungen ohne Körperkontakt

**Abb. 1:**
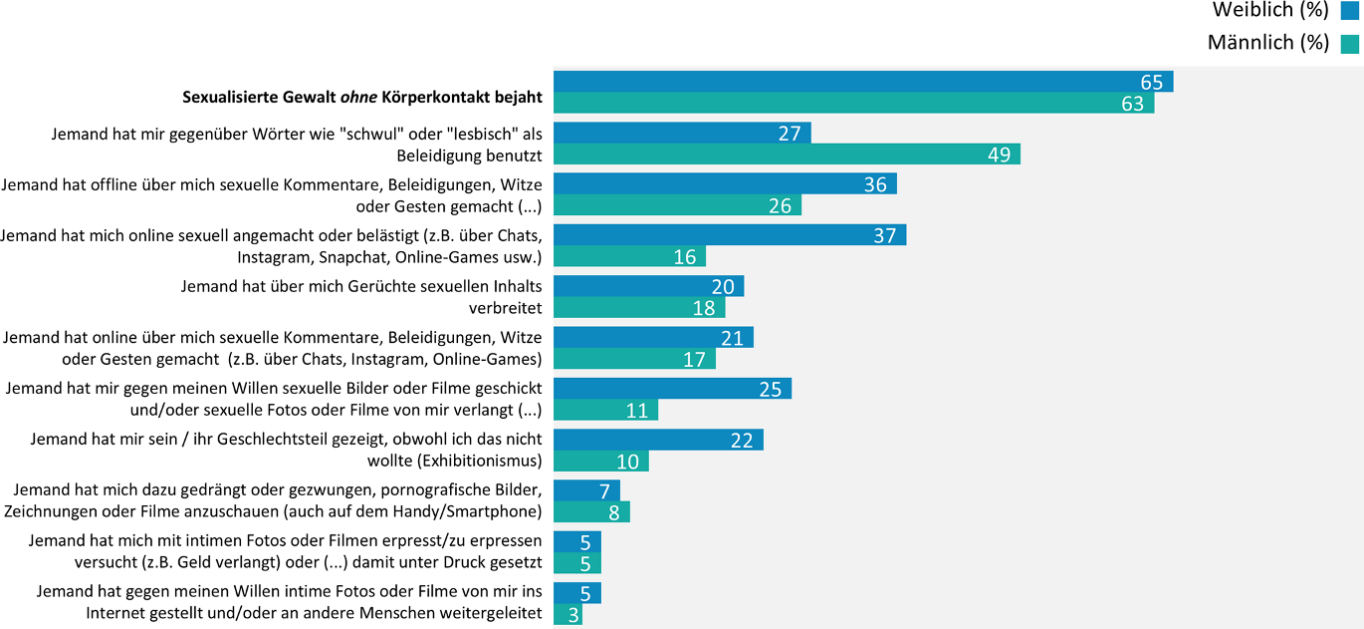
Sexualisierte Gewalt ohne Körperkontakt bei Jugendlichen und jungen Erwachsenen nach Geschlecht, Ergebnisse der 10. Welle der Jugendsexualitätsstudie 2025. Frage: Haben Sie solche oder ähnliche Dinge selbst schon mal erlebt? Basis: alle Befragten (*N* = 5855) | Mehrfachnennungen möglich | Darstellung: Genannt-Anteile in Prozent

Insgesamt berichten 64 % der befragten Jugendlichen und jungen Erwachsenen, mindestens einmal eine Form sexualisierter Gewalt ohne Körperkontakt erlebt zu haben, wobei der Anteil bei den jungen Erwachsenen höher liegt als bei den Jugendlichen (68 % gegenüber (ggü.) 54 %). Männliche und weibliche Befragte sind insgesamt in etwa gleich häufig betroffen (63 % ggü. 65 %), allerdings unterscheiden sich die erlebten Gewaltformen (Abb. 1).

Zudem zeigt sich, dass Personen mit homosexueller oder bisexueller Orientierung deutlich häufiger von sexualisierter Gewalt *ohne *Körperkontakt betroffen sind als Befragte mit heterosexueller Orientierung (90 % ggü. 61 %).^4^

#### Sexualisierte Gewalterfahrungen mit Körperkontakt

**Abb. 2:**
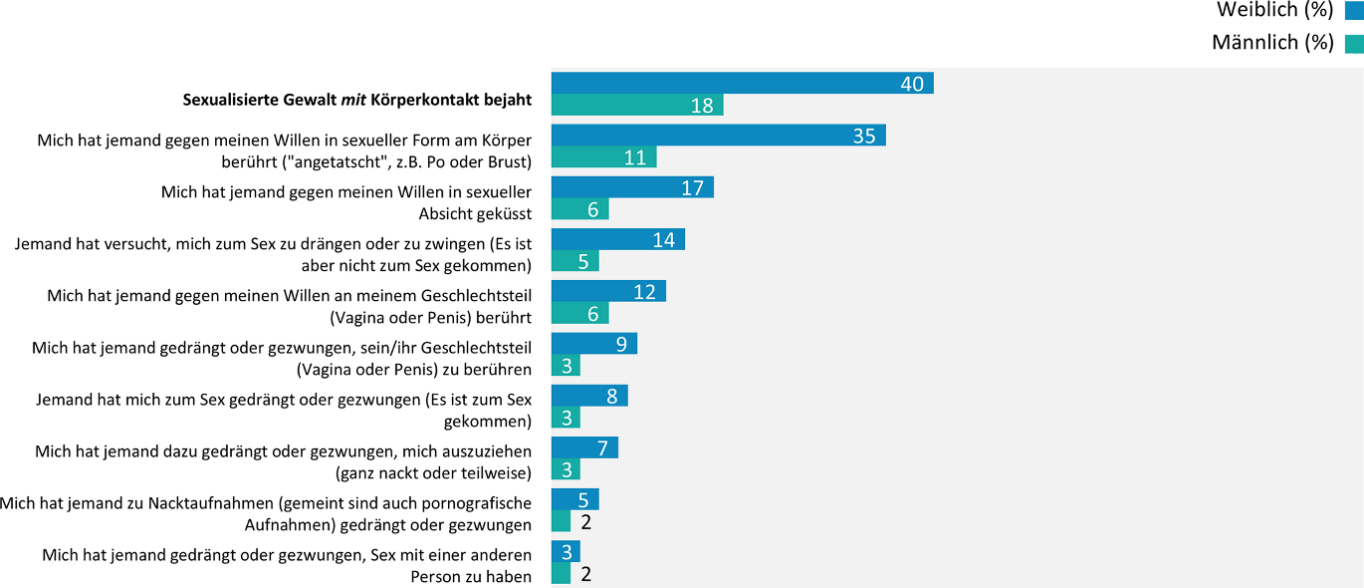
Sexualisierte Gewalt mit Körperkontakt bei Jugendlichen und jungen Erwachsenen nach Geschlecht, Ergebnisse der 10. Welle der Jugendsexualitätsstudie 2025. Frage: Und wie ist es hiermit? Haben Sie solche oder ähnliche Dinge selbst schon mal erlebt? Basis: alle Befragten (*N* = 5855) | Mehrfachnennungen möglich | Darstellung: Genannt-Anteile in Prozent

Sexualisierte Gewalt *mit* Körperkontakt hat knapp ein Drittel (29 %) der Befragten mindestens einmal in seinem Leben erfahren (Jugendliche: 12 %, junge Erwachsene: 37 %). Weibliche Personen sind doppelt so häufig betroffen wie männliche Personen (40 % ggü. 18 %), unabhängig der abgefragten Situation bzw. Handlung (Abb. 2).

Häufig betroffen sind außerdem die Befragten, die sich als homo- und/oder bisexuell identifizieren (57 % ggü. 26 %).^4^

### Alter der betroffenen Personen bei der ersten sexualisierten Gewalterfahrung mit Körperkontakt

Die Mehrheit der betroffenen Personen war zum Tatzeitpunkt minderjährig (54 %), wobei rund 15 % jünger als 14 Jahre alt waren. 58 % der Betroffenen waren älter als 14 Jahre, wobei lediglich 20 % bereits volljährig waren. Über ein Viertel der befragten Personen (27 %) macht keine Angabe hinsichtlich ihres Alters zum Zeitpunkt der Tat.

### Merkmale der ausübenden Personen bei der ersten sexualisierten Gewalterfahrung mit Körperkontakt

Meist wurde die sexualisierte Gewalt mit Körperkontakt von einer Person ausgeübt (69 %), nur selten waren 2 oder mehr Personen beteiligt (2 Personen: 6 %, mehr als 2 Personen: 3 %). In den meisten Fällen handelt es sich um Jugendliche (45 %) oder Erwachsene (34 %).

Insgesamt sind 71 % der ausübenden Personen männlich, 11 % weiblich und in 2 % der Fälle handelt es sich um sowohl männliche als auch weibliche Personen. Es zeigt sich hierbei eine deutliche Abhängigkeit des Geschlechts der ausübenden Person zum Geschlecht der betroffenen Person: 89 % der weiblichen Betroffenen geben an, dass die ausübende(n) Person(en) männlich war(en), bei den männlichen Betroffenen waren es 34 %. Männliche Betroffene geben deutlich häufiger an, dass die Tat durch eine/mehrere weibliche Person(en) ausgeübt wurde (34 %), während dies nur von 1 % der betroffenen Mädchen und jungen Frauen angegeben wird. Männliche Betroffene machten insgesamt jedoch auch deutlich häufiger keine Angabe zum Geschlecht der ausübenden Person(en) (28 % ggü. 9 % bei weiblichen Betroffenen).

Bei den ausübenden Personen handelt es sich zumeist um Freund(innen), Mitschüler(innen) oder Kolleg(innen) (28 %), aber auch um unbekannte Personen (26 %). In 18 % der Fälle haben sich neue Bekanntschaften (zum Beispiel aus der Disco) körperlich übergriffig verhalten, in 13 % der Fälle war es der/die Freund(in) bzw. Exfreund(in) aus einer festen Beziehung. Seltener genannt werden hingegen Personen aus der Familie bzw. Verwandtschaft (6 %) oder der Nachbarschaft (5 %) sowie Personen, zu denen ein Abhängigkeitsverhältnis besteht, wie beispielsweise Betreuungspersonen (2 %).

Die Beziehung zur ausübenden Person ist maßgeblich mit dem Alter der betroffenen Person assoziiert: Waren die betroffenen Personen zum Zeitpunkt der Tat jünger als 14 Jahre, geben sie eher an, dass jemand aus der Familie oder Verwandtschaft die Tat ausgeübt hat (24 % ggü. 2 % 14 Jahre und älter). Ebenso geben sie häufiger an, dass sich eine Person aus der Nachbarschaft körperlich übergriffig verhalten hat (13 % ggü. 5 %). War die betroffene Person zum Tatzeitpunkt jedoch 14 Jahre oder älter, werden eher der/die Freund(in) bzw. Exfreund(in) aus einer festen Beziehung (17 % ggü. 5 % jünger als 14 Jahre) sowie neue Bekanntschaften angegeben (22 % ggü. 10 %).

**Abb. 3:**
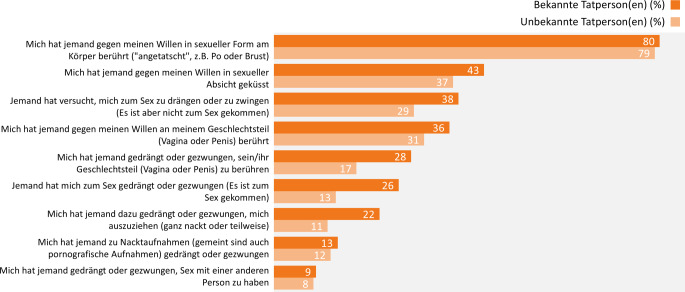
Sexualisierte Gewalt mit Körperkontakt bei Jugendlichen und jungen Erwachsenen: Gewalterfahrungen in Abhängigkeit der Bekanntheit der Person, Ergebnisse der 10. Welle der Jugendsexualitätsstudie 2025. Frage: Und wie ist es hiermit? Haben Sie solche oder ähnliche Dinge selbst schon mal erlebt? Basis: körperliche sexualisierte Gewalterfahrung bejaht (*n* = 1440) | Mehrfachnennungen möglich | Darstellung: Genannt-Anteile in Prozent

Auffällig ist des Weiteren, dass sich die Art der erlebten sexualisierten Gewalt in Abhängigkeit vom Bekanntheitsgrad der ausübenden Person unterscheidet (Abb. 3). Es ist dabei zu berücksichtigen, dass Mehrfachangaben hinsichtlich der Gewalterfahrungen möglich waren, die Angaben über die ausübende(n) Person(en) sich jedoch auf die erste erlebte Tat bezogen.

### Sexualisierte Gewalt unter Einbezug intimer Fotos und Videos

Insgesamt gibt ein Viertel der Befragten (24 %) an, sexualisierte Gewalt erlebt zu haben, bei denen intime Fotos oder Videos eingesetzt wurden (Tab. 1). Personen mit homosexueller oder bisexueller Orientierung berichten mehr als doppelt so häufig von entsprechenden Erlebnissen wie Personen mit (überwiegend) heterosexueller Orientierung (54 % ggü. 21 %). Etwa 2 von 10 der Befragten geben an, dass sie bereits gegen ihren Willen intime Fotos bzw. Videos erhalten haben oder dass diese von ihnen verlangt wurden. Weibliche Befragte sind hiervon deutlich häufiger betroffen.

**Tab. 1:** Häufigkeit sexualisierter Gewalt unter Beteiligung intimer Fotos und Videos in Abhängigkeit der sexuellen Orientierung bei Jugendlichen und jungen Erwachsenen (*N* *=* 5855). Ergebnisse der 10. Welle der Jugendsexualitätsstudie 2025. *n* entspricht der ungewichteten Fallzahl; Mehrfachnennungen möglich.

Sexualisierte Gewalt unter Beteiligung intimer Fotos oder Videos	Gesamt		Sexuelle Orientierung: mind. vorwiegend …	
			Heterosexuell	Homosexuell/bisexuell
	*n*	%	%	%
Jemand hat mir gegen meinen Willen sexuelle Bilder oder Filme geschickt und/oder sexuelle Fotos oder Filme von mir verlangt …	916	18	15	45
Jemand hat mich dazu gedrängt oder gezwungen, pornografische Bilder, Zeichnungen oder Filme anzuschauen (auch auf dem Handy/Smartphone)	407	8	7	19
Jemand hat mich mit intimen Fotos oder Filmen erpresst/zu erpressen versucht (z. B. Geld verlangt) oder … damit unter Druck gesetzt	214	5	4	11
Jemand hat gegen meinen Willen intime Fotos oder Filme von mir ins Internet gestellt und/oder an andere Menschen weitergeleitet	195	4	3	12
Mich hat jemand zu Nacktaufnahmen (gemeint sind auch pornografische Aufnahmen) gedrängt oder gezwungen	171	4	3	11

Andere Einsatzformen intimer Fotos und Videos im Kontext sexualisierter Gewalt treten zwar erheblich seltener auf, jedoch geben insgesamt 13 % an, eine dieser Formen erfahren zu haben (Tab. 1). Weibliche und männliche Befragte sind hierbei ähnlich häufig betroffen (Abweichungen zwischen 1 bis 3 Prozentpunkte).

### Sexualisierte Gewalt im Beisein bzw. mit Kenntnis von Bystandern

#### Bystander-Perspektive aus Sicht aller Befragten

38 % der Jugendlichen und jungen Erwachsenen berichten, schon einmal mitbekommen zu haben, dass eine andere Person zu sexuellen Handlungen gedrängt oder gezwungen wurde. In den meisten Fällen erfahren die Befragten durch die betroffene Person (25 %) von der Tat oder durch andere, die die Tat mitbekommen haben (15 %). Selten waren die Befragten selbst bei der Tat anwesend (7 %) oder haben davon durch die ausübende Person erfahren (1 %).

Ob Personen schon einmal eine Bystander-Perspektive eingenommen haben, steht unter anderem in Zusammenhang mit ihrem Geschlecht sowie ihrer sexuellen Orientierung. So berichten weibliche Befragte häufiger als männliche Befragte, bereits Bystander gewesen zu sein (42 % ggü. 34 %), vor allem durch entsprechende Offenlegungen der betroffenen Personen (weiblich: 31 % ggü. männlich: 18 %). Ebenso geben homo- und bisexuelle Befragte häufiger als heterosexuelle Befragte an, schon einmal eine Bystander-Perspektive eingenommen zu haben (54 % ggü. 36 %), was ebenfalls vorwiegend auf die Offenlegung durch die betroffenen Personen zurückzuführen ist (homo-/bisexuell: 44 % ggü. heterosexuell: 22 %).

#### Anwesenheit von Bystandern bei der ersten erlebten sexualisierten Gewalt mit Körperkontakt

**Abb. 4:**
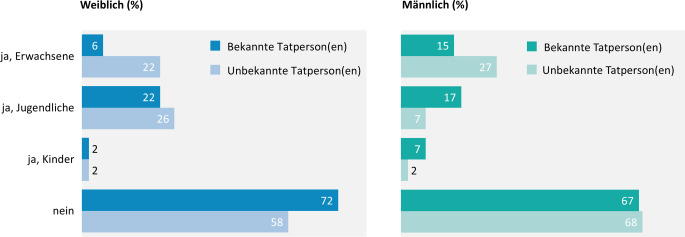
Sexualisierte Gewalt mit Körperkontakt bei Jugendlichen und jungen Erwachsenen: Anwesenheit von Bystandern in Abhängigkeit der Bekanntheit der Tatperson und nach Geschlecht, Ergebnisse der 10. Welle der Jugendsexualitätsstudie 2025. Frage: Waren andere/weitere Personen außer den Tatpersonen in der konkreten Situation in der Nähe oder haben etwas davon mitbekommen? Basis: körperliche sexualisierte Gewalterfahrung bejaht (*n* = 1440) | Mehrfachnennungen möglich | Darstellung: Genannt-Anteile in Prozent

Bei 31 % der ersten sexualisierten Gewalterfahrung mit Körperkontakt sind Bystander während der Tat anwesend. Dabei handelt es sich vor allem um Jugendliche (19 %) oder Erwachsene (14 %), während Kinder insgesamt nur selten Zeugen der Tat werden (3 %). Letzteres ist eher dann der Fall, wenn die betroffenen Personen zum Tatzeitpunkt selbst Kinder sind, also jünger als 14 Jahre (13 % ggü. 2 % 14 Jahre und älter). Die Anwesenheit von Bystandern wird unterschiedlich häufig angegeben, je nachdem, ob die ausübenden Personen den Befragten bekannt oder unbekannt sind (Abb. 4).

### Disclosure nach der ersten erlebten sexualisierten Gewalt mit Körperkontakt

#### Einflussfaktoren auf den Disclosure-Prozess

Insgesamt vertrauen sich 69 % der Betroffenen nach der ersten körperlichen Gewalterfahrung mindestens einer anderen Person an. Zumeist geschieht dies direkt nach der Tat (32 %), hingegen seltener Tage (14 %), Wochen bzw. Monate (11 %) oder Jahre (12 %) später. Etwa ein Viertel der Betroffenen hat das Erlebte niemandem offengelegt (24 %).

**Abb. 5:**
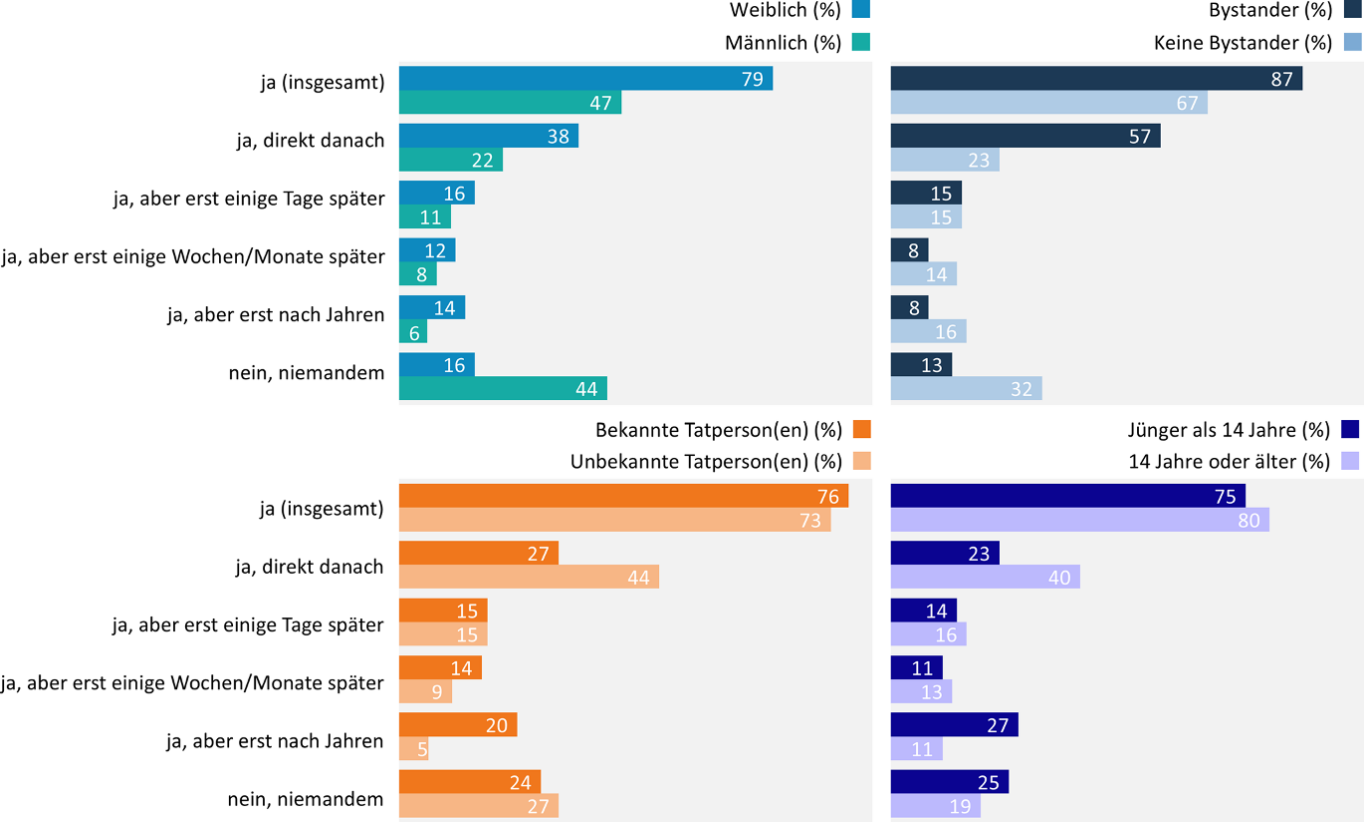
Sexualisierte Gewalt mit Körperkontakt bei Jugendlichen und jungen Erwachsenen: Zeitpunkt der Offenlegung in Abhängigkeit von Geschlecht, Anwesenheit von Bystandern, Bekanntheit der Tatperson(en) und Alter zum Tatzeitpunkt. Ergebnisse der 10. Welle der Jugendsexualitätsstudie 2025. Frage: Haben Sie jemandem von diesem Erlebnis erzählt? Basis: körperliche sexualisierte Gewalterfahrung bejaht (*n* = 1440) | Mehrfachnennungen möglich | Darstellung: Genannt-Anteile in Prozent

Ob und zu welchem Zeitpunkt die Tat offenbart wird, steht unter anderem im Zusammenhang mit dem Geschlecht der betroffenen Person, der Anwesenheit von Bystandern während der Tat, dem Bekanntheitsgrad der ausübenden Person(en) sowie dem Alter der betroffenen Person (Abb. 5).

Als häufigste Gründe für eine Nichtoffenlegung des Erlebten benennen betroffene Personen, dass sie „es nicht so schlimm fanden“ (29 %), dass sie „selbst nicht mehr daran denken wollten“ (26 %) und dass sie „sich schämten“ (24 %). Männliche Befragte führen dabei etwas häufiger als weibliche Befragte an, dass sie die Tat „nicht so schlimm fanden“ (34 % ggü. 22 %). Weibliche Befragte nennen als Gründe hingegen häufiger, dass sie „nicht mehr daran denken wollten“ (40 % ggü. 17 %) bzw. sich nach eigener Aussage schämten (36 % ggü. 15 %).

#### Adressierte im Disclosure-Prozess

Wenn die betroffenen Personen die erste sexualisierte Gewalterfahrung mit Körperkontakt jemandem anvertrauen, spielen insbesondere Gleichaltrige eine wichtige Rolle. So werden Freund(innen) in 70 % der Fälle, der/die damalige Freund(in) in 16 % der Fälle adressiert. Etwa die Hälfte (51 %) der betroffenen Personen vertraut sich ausschließlich Gleichaltrigen an.

Doch auch die eigenen Eltern sind wichtige Ansprechpersonen (33 %) – im Gegensatz zu Fachkräften wie Therapeut(innen) (10 %), Ärzt(innen) (2 %), Lehrkräften (5 %), Polizei (3 %) oder anderen Erwachsenen (10 %). Ob den eigenen Eltern das Erlebte anvertraut wird, hängt damit zusammen, inwiefern im eigenen Elternhaus offen über Themen wie Sexualität und Verhütung gesprochen wird. Ist dies der Fall, erfolgt eine Offenlegung deutlich häufiger als in Fällen, in denen diese Themen nicht angesprochen werden (Sexualität: 40 % ggü. 20 %; Verhütung: 45 % ggü. 21 %).

**Abb. 6:**
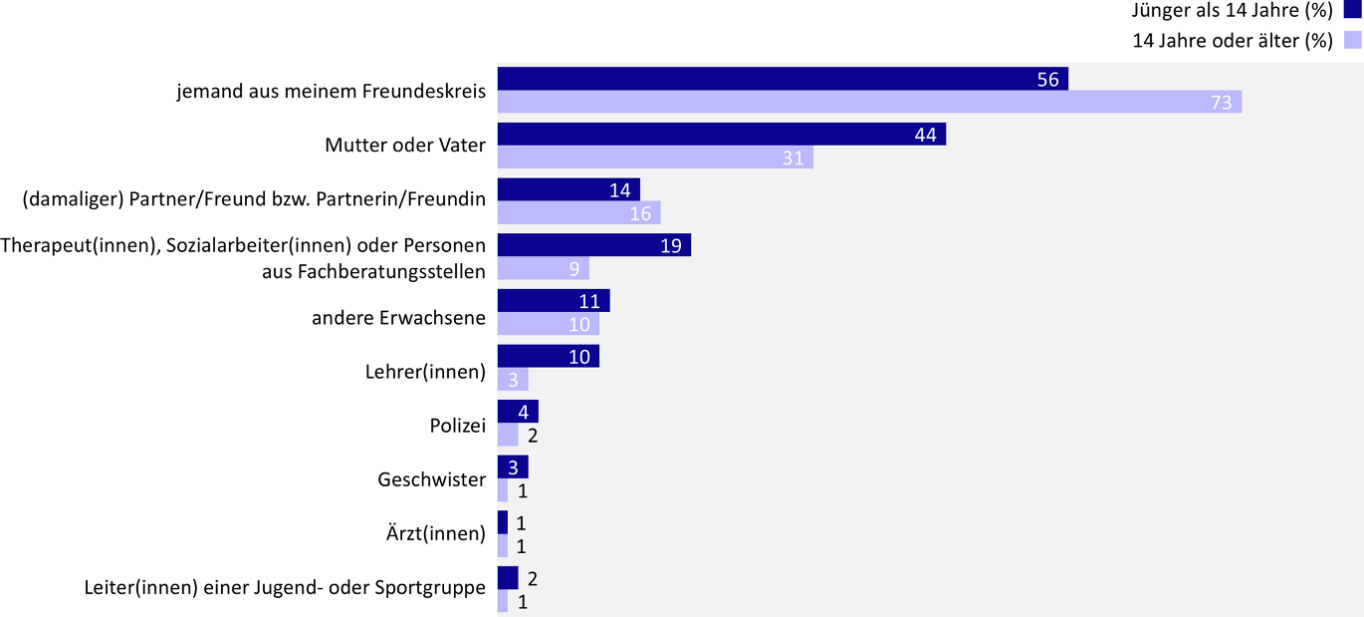
Sexualisierte Gewalt mit Körperkontakt bei Jugendlichen und jungen Erwachsenen: Adressierte im Disclosure-Prozess in Abhängigkeit vom Alter zum Tatzeitpunkt, Ergebnisse der 10. Welle der Jugendsexualitätsstudie 2025. Frage: Wem haben Sie davon erzählt? Basis: Offenlegung über Erlebnis körperlicher sexualisierter Gewalterfahrung bejaht (*n* = 1039) | Mehrfachnennungen möglich | Darstellung: Genannt-Anteile in Prozent

Des Weiteren hat das Alter der betroffenen Person zum Tatzeitpunkt Einfluss darauf, wer im Disclosure-Prozess adressiert wird (Abb. 6). So vertrauen sich Personen, die bei der Tat jünger als 14 Jahre alt waren, eher ihren Eltern an als Personen, die zum Tatzeitpunkt älter waren. Zudem wenden sich Jüngere deutlich häufiger an professionelle Ansprechpersonen wie Therapeut(innen), Sozialarbeiter(innen) oder Personen aus Fachberatungsstellen. Waren die Betroffenen hingegen bei der Tat älter als 14 Jahre, treten sie eher an jemanden aus ihrem Freundeskreis heran.

#### Bewertung des Disclosure-Prozesses

Personen, die die erste sexualisierte Gewalterfahrung mit Körperkontakt jemandem anvertrauen, empfinden das Gespräch überwiegend als „etwas“ bis „sehr hilfreich“ (85 %), während etwa 14 % das Gespräch als „wenig“ bis „gar nicht hilfreich“ bewerten. Dabei zeigen sich keine Geschlechterunterschiede. Auch ob die ausübende Person bekannt oder unbekannt ist, spielt für die Bewertung des Disclosure-Prozesses keine Rolle. Wesentlich ausschlaggebender ist hingegen das Alter der betroffenen Person zum Zeitpunkt der Tat: Personen, die bei der Gewalterfahrung jünger als 14 Jahre alt waren, erleben das Gespräch als deutlich weniger hilfreich als Personen, die bei der Tat älter waren (69 % ggü. 88 %).

## Diskussion

Die Ergebnisse der vorliegenden Studie belegen die hohe Betroffenheit junger Menschen von sexualisierter Gewalt. Rund zwei Drittel der befragten Jugendlichen und jungen Erwachsenen hat mindestens eine Form sexualisierter Gewalt *ohne* Körperkontakt erlebt, wiederum knapp ein Drittel mindestens eine Form sexualisierter Gewalt *mit* Körperkontakt. Jede vierte Person hat den Einsatz von intimem Bild- und Videomaterial bei der Ausübung sexualisierter Gewalt erfahren, insbesondere den Erhalt oder das Verlangen intimer Fotos bzw. Videos gegen ihren Willen. Bei 45 % der ausübenden Personen der ersten Gewalterfahrung *mit* Körperkontakt handelt es sich um Jugendliche.

Bezogen auf die Bystander-Perspektive berichten knapp 40 % der befragten Jugendlichen und jungen Erwachsenen, schon einmal mitbekommen zu haben, wie andere zu sexuellen Handlungen gedrängt oder gezwungen wurden. In den meisten Fällen haben sie es dadurch mitbekommen, dass ihnen jemand davon erzählt hat, nur selten waren die Personen selber anwesend. Etwa ein Drittel der ersten Gewalterfahrungen *mit* Körperkontakt geschah im Beisein bzw. mit Kenntnis von Dritten und etwa die Hälfte der Betroffenen vertraut sich danach ausschließlich Gleichaltrigen an.

Die Ergebnisse der 10. Trendwelle der Jugendsexualitätsstudie bestätigen in weiten Teilen die Befunde nationaler und internationaler Untersuchungen, wonach das Jugendalter eine Phase erhöhten Risikos ist, sexualisierte Gewalt zu erleben [1–11, 16, 19, 22, 25]. Dies betrifft sowohl körperliche als auch nichtkörperliche Gewalterfahrungen. Der digitale Raum als Tatkontext [2, 31–36] wurde in der vorliegenden Studie nicht explizit in den Blick genommen. Um aber einen Einblick in die Relevanz digitaler Medien bei Erfahrungen sexualisierter Gewalt zu erhalten, wurde eine separate Auswertung entsprechender Items durchgeführt. Es zeigt sich eine hohe Betroffenheit insbesondere von weiblichen sowie homo- und bisexuellen Personen. Hierbei geht es um zum Teil strafrechtlich hochrelevante Taten wie das nicht einvernehmliche Weiterleiten von oder das Erpressen mit intimen Fotos bzw. Filmen.

Die Befunde unterstreichen außerdem, dass Jugendliche – anders als in der Kindheit – sexualisierte Gewalt häufig durch Jugendliche in einem ähnlichen Alter in ihrem sozialen Nahfeld erfahren (Peer-Gewalt; [1, 2, 4, 6–10, 12–23, 37]). In diesem Zusammenhang verweisen internationale und zuletzt auch vermehrt nationale Studien auf die Bedeutung von Gruppenprozessen unter Jugendlichen, die sexualisierte Gewalt verstärken, aber auch verhindern können [9, 14, 15, 17, 23].

Wie in anderen Studien mittlerweile vielfach belegt, findet sexualisierte (Peer‑)Gewalt häufig in Anwesenheit oder mit Kenntnis jugendlicher Bystander statt [4, 8, 12, 21, 23, 24, 37]. In der vorliegenden Befragung wurde dies bestätigt (s. oben). Die Studie gibt Hinweise darauf, dass Bystander auch im digitalen Raum direkt oder indirekt miteingebunden werden, wenn beispielsweise das Teilen und Weiterleiten intimen Bild- und Videomaterials angedroht bzw. umgesetzt wird.

Auch die Offenlegung erfolgt überwiegend, häufig auch ausschließlich, unter Gleichaltrigen [4, 8, 9, 16, 26]. Die vorliegenden Daten bestätigen, dass aus Sicht der betroffenen Personen hilfreiche Disclosure-Prozesse hoch voraussetzungsreich sind [2, 4, 8, 9, 16, 26, 38], insbesondere dann, wenn die Gewalt in ihrem unmittelbaren familiären oder sozialen Umfeld stattfand. Während Fachkräfte auch in dieser Studie selten adressiert werden [4, 8], sind Eltern durchaus wichtige Ansprechpersonen. Es zeigt sich, dass ein offener Umgang mit den Themen Sexualität und Verhütung im Elternhaus einen großen Einfluss auf den Disclosure-Prozess von Betroffenen hat. Dies beweist einmal mehr das enge Zusammenspiel von sexueller Bildung und der Prävention sexualisierter Gewalt [28, 39–43].

### Limitationen

Die vorliegende Befragung liefert eine umfangreiche repräsentative Datenlage zur Erfahrung sexualisierter Gewalt von jungen Menschen, die einer sorgsamen Planung und einem methodisch-fundierten Vorgehen unterliegt. Jedoch sind limitierende Aspekte anzuführen.

Erstens wurden nichtkörperliche Gewalterfahrungen weniger detailliert erfasst als körperliche. In der Konsequenz wurde auch die Rolle von Bystandern bei nichtkörperlichen Gewalterfahrungen nicht in gleicher Weise untersucht. Andere Studien legen jedoch nahe, dass insbesondere im Jugendalter nichtkörperliche Gewalterfahrungen häufig im Beisein bzw. mit Kenntnis anderer Peers stattfinden [8, 24].

Zweitens sind aufgrund der Querschnittserhebung Aussagen über Kausalzusammenhänge nicht möglich sowie Teilanalysen teils durch kleine Fallzahlen limitiert, so zum Beispiel bei der Betroffenheit im Alter unter 14 Jahren sowie bezogen auf Gewalterfahrungen von homo- und bisexuellen jungen Menschen. Auch konnten Personen außerhalb der binären Geschlechterkategorien mangels amtlicher Referenzzahlen noch nicht separat gewichtet werden. An dieser Stelle sei auf verschiedene explorative Studien verwiesen, die wichtige Erkenntnisse zu sexuellen und partnerschaftlichen Erfahrungen von LSBTIQ*-Personen (Lesben, Schwule, Bisexuelle, Trans*, Inter*, Queers und nicht benannte Identitäten) sowie zu ihren (fehlenden) Zugangsmöglichkeiten zu Beratungsangeboten generieren (siehe auch Beitrag von Stehr und Wazlawik in diesem Themenheft) [44–46].

Drittens können mögliche Verzerrungen im Antwortverhalten der Jugendlichen und jungen Erwachsenen (z. B. Social Desirability Bias) trotz des Selbstausfüllerteils bei Fragen zu sexualisierten Gewalterfahrungen nicht vollständig ausgeschlossen werden [44]. Ein Bias im Antwortverhalten ist unter anderem dann zu erwarten, wenn sexualisierte Gewalt im familiären Setting erlebt wird/wurde, da die Interviews bei Minderjährigen nur mit Einverständnis der Erziehungsberechtigten stattfanden und sich diese währenddessen, wenn auch nicht direkt im Raum, so doch in der Regel im selben Haushalt aufhielten [45]. Gleiches wäre zu erwarten, wenn die Befragung im Setting Schule oder Freizeit stattfände, da Peers erwiesenermaßen bei Gewalterfahrungen insbesondere im Jugendalter eine wichtige Rolle spielen [45].

## Fazit

Die 10. Welle der Jugendsexualitätsstudie liefert aktuelle Daten zu sexualisierten Gewalterfahrungen bei Jugendlichen und jungen Erwachsenen. Erste Ergebnisse der Studie zur Verbreitung körperlicher und nichtkörperlicher sexualisierter Gewaltformen sowie die Rolle von Bystandern währenddessen und im Disclosure-Prozess sind hier auf deskriptiver Ebene dargestellt (eine inferenzstatistische Betrachtung erfolgt an anderer Stelle). Trotz oben genannter Limitationen leisten die vorliegenden Daten damit einen wertvollen Beitrag, um junge Menschen, Eltern und Fachkräfte bei der Aufklärungs- und Präventionsarbeit zu unterstützen.

Während für Kinder der Schutz vor Viktimisierungen durch Erwachsene und die Begleitung durch Vertrauenspersonen wie Fachkräfte im Vordergrund stehen, sollten Maßnahmen zur Prävention sexualisierter (Peer‑)Gewalt an der Lebenswelt der Jugendlichen ansetzen und dabei alle drei Perspektiven berücksichtigen: die Perspektive der betroffenen Person(en), der tatausübenden Person(en) sowie der Bystander. Das BIÖG hat 2025 den gesetzlichen Auftrag erhalten, bundesweit die Prävention und den Schutz von Kindern und Jugendlichen vor sexueller Gewalt und Ausbeutung auszubauen.^5^

## Supplementary Information

ESM1: Zusatzmaterial 1

## Data Availability

Die im Rahmen dieser Studie erhobenen Daten werden voraussichtlich über GESIS (Leibniz-Institut für Sozialwissenschaften) veröffentlicht, um die Ergebnisse der Wissenschaft zugänglich zu machen.

## Footnotes

1 Im Februar 2025 wurde die ehemalige Bundeszentrale für gesundheitliche Aufklärung (BZgA) umbenannt in Bundesinstitut für Öffentliche Gesundheit (BIÖG).

2 Zentrale Ergebnisse der Jugendsexualitätsstudie 2025 zum Sexual- und Verhütungsverhalten finden sich im Beitrag von Scharmanski und Dinger in diesem Themenheft.

3 Die Instruktionen und Itemformulierungen können in Tabelle Z1 im Onlinematerial eingesehen werden.

4 Basis: *n* = 418 mind. vorwiegend homosexuell/bisexuell orientiert und *n* = 5313 mind. vorwiegend heterosexuell orientiert, ungewichtet.

5 Siehe Pressemitteilung vom 31.01.2025 des BIÖG (vormals BZgA): https://www.bioeg.de/presse/pressemitteilungen/2025-01-31-kinder-besser-schuetzen-bzga-erhaelt-gesetzlichen-auftrag-zur-praevention-sexueller-gewalt-an-kindern-und-jugendlichen/.
