# Supplementary material for: Experiences of sexual violence, bystander perspectives, and disclosure among young people—results of the 10th wave of the representative survey “Youth Sexuality”
Source: Bundesgesundheitsblatt Gesundheitsforschung Gesundheitsschutz. 2026 Mar 27;69(4):434–43. [Article in German] doi: 10.1007/s00103-026-04212-y (PMC13043535; doi:10.1007/s00103-026-04212-y)
Supplement: Supplementary file 1 — ESM1: Zusatzmaterial 1 [file 103_2026_4212_MOESM1_ESM.pdf]

Bundesinstitut für Öffentliche Gesundheit

Tabelle Z1: Fragestellungen, Antwortmöglichkeiten und Datenbasis zu Erfahrungen sexualisierter Gewalt bei Jugendlichen und jungen Erwachsenen in der 10. Befragungswelle der Jugendsexualitätsstudie 2025

**Erfahrungen sexualisierter Gewalt ohne und mit Körperkontakt**		
**Fragestellungen**	**Antwortmöglichkeiten**	**Datenbasis**
Haben Sie solche oder ähnliche Dinge selbst schon mal erlebt?<br><br>*(angelehnt an die SPEAK-Studie, vgl. Maschke/Stecher 2018*)*	Mehrfachnennungen möglich<br><br>(1) Jemand hat online über mich sexuelle Kommentare, Beleidigungen, Witze oder Gesten gemacht (z. B. über Chats, Instagram, Online-Games)<br>(2) Jemand hat offline über mich sexuelle Kommentare, Beleidigungen, Witze oder Gesten gemacht (z. B. auf der Straße, in der Schule, am Arbeitsplatz)<br>(3) Jemand hat mir sein/ihr Geschlechtsteil gezeigt, obwohl ich das nicht wollte (Exhibitionismus)<br>(4) Jemand hat mich dazu gedrängt oder gezwungen, pornografische Bilder, Zeichnungen oder Filme anzuschauen (auch auf dem Handy/Smartphone)<br>(5) Jemand hat mich online sexuell angemacht oder belästigt (z. B. über Chats, Instagram, Snapchat, Online-Games usw.)<br>(6) Jemand hat gegen meinen Willen intime Fotos oder Filme von mir ins Internet gestellt und/oder an andere Menschen weitergeleitet<br>(7) Jemand hat mir gegenüber Wörter wie „schwul“ oder „lesbisch“ als Beleidigung benutzt<br>(8) Jemand hat über mich Gerüchte sexuellen Inhalts verbreitet<br>(9) Jemand hat mich mit intimen Fotos oder Filmen erpresst/zu erpressen versucht (z. B. Geld verlangt) oder auf andere Weise damit unter Druck gesetzt<br>(10) Jemand hat mir gegen meinen Willen sexuelle Bilder oder Filme geschickt und/oder sexuelle Fotos oder Filme von mir verlangt (z. B. Fotos vom Geschlechtsteil)<br><br>1: ja<br>2: nein<br><br>9: keine Angabe	Alle Befragten<br>(n = 5.855, davon n = 3.556 weiblich, n = 2.256 männlich und n = 43 ohne Geschlechtszuordnung) bzw.<br><br>n = 3.514 14- bis 17-jährige Jugendliche und n = 2.341 18- bis 25-jährige junge Erwachsene bzw.<br><br>n = 418 mind. vorwiegend homosexuell/bisexuell orientiert und n = 5.313 mind. vorwiegend heterosexuell orientiert, ungewichtet)

Bundesinstitut für Öffentliche Gesundheit

<table>
<tr>
<td>Und wie ist es hiermit? Haben Sie solche oder ähnliche Dinge selbst schon mal erlebt?

(angelehnt an die SPEAK-Studie, vgl. Maschke/Stecher 2018*)</td>
<td>Mehrfachnennungen möglich

(1) Mich hat jemand dazu gedrängt oder gezwungen, mich auszuziehen (ganz nackt oder teilweise)
(2) Mich hat jemand gegen meinen Willen an meinem Geschlechtsteil (Vagina oder Penis) berührt
(3) Mich hat jemand gedrängt oder gezwungen, sein/ihr Geschlechtsteil (Vagina oder Penis) zu berühren
(4) Mich hat jemand gegen meinen Willen in sexueller Absicht geküsst
(5) Mich hat jemand gedrängt oder gezwungen, Sex mit einer anderen Person zu haben
(6) Mich hat jemand zu Nacktaufnahmen (gemeint sind auch pornografische Aufnahmen) gedrängt oder gezwungen
(7) Jemand hat versucht, mich zum Sex zu drängen oder zu zwingen (Es ist aber nicht zum Sex gekommen)
(8) Jemand hat mich zum Sex gedrängt oder gezwungen (Es ist zum Sex gekommen)
(9) Mich hat jemand gegen meinen Willen in sexueller Form am Körper berührt („angetatscht“, z. B. Po oder Brust)

1: ja
2: nein

9: keine Angabe</td>
<td>Alle Befragten

(n = 5.855, davon n = 3.556 weiblich, n = 2.256 männlich und n = 43 ohne Geschlechtszuordnung bzw.

n = 3.514 14- bis 17-jährige Jugendliche und n = 2.341 18- bis 25-jährige junge Erwachsene bzw.

n = 418 mind. vorwiegend homosexuell/bisexuell orientiert und n = 5.313 mind. vorwiegend heterosexuell orientiert, ungewichtet)</td>
</tr>
<tr>
<td>Wie alt waren Sie, als das das erste Mal passierte?

Wenn es mehrere dieser Situationen gab, denken Sie bitte bei den folgenden Fragen an das erste Mal, als jemand versucht hat, Sie gegen Ihren Willen zu körperlichen Berührungen oder sexuellen Handlungen zu bringen.</td>
<td>Offene Nennung</td>
<td>14- bis 25-Jährige, die sexualisierte Gewalt mit Körperkontakt bejahten (n = 1.440, ungewichtet)</td>
</tr>
</table>

**Angaben zu Tatpersonen**		
Wie viele Tatpersonen waren an der Tat beteiligt?	1: eine Person<br>2: zwei Personen<br>3: mehr als zwei Personen<br><br>8: weiß nicht (mehr)<br>9: keine Angabe	14-bis 25-Jährige, die sexualisierte Gewalt mit Körperkontakt bejahten (n = 1.440, ungewichtet)
(Eine Tatperson)<br>Wie alt war die Tatperson ungefähr?<br><br>(Mehrere Tatpersonen)<br>Wie alt waren die Tatpersonen ungefähr?	(Eine Tatperson)<br>1: Kind<br>2: jugendlich<br>3: erwachsen<br><br>8: weiß nicht (mehr)<br>9: keine Angabe<br><br>(Mehrere Tatpersonen, Mehrfachnennungen möglich)<br>11: Kind(er)<br>12: jugendlich<br>13: erwachsen<br><br>98: weiß nicht (mehr)<br>99: keine Angabe	14-bis 25-Jährige, die sexualisierte Gewalt mit Körperkontakt bejahten (n = 1.440, ungewichtet)
(Eine Tatperson)<br>War die Tatperson…?<br><br>(Mehrere Tatpersonen)<br>Waren die Tatpersonen…?	(Eine Tatperson)<br>1: männlich<br>2: weiblich<br><br>(nur bei Angabe mehrerer Tatpersonen)<br>3: männlich und weiblich<br><br>8: weiß nicht (mehr)<br>9: keine Angabe	14-bis 25-Jährige, die sexualisierte Gewalt mit Körperkontakt bejahten (n = 1.440, davon n = 1.121 weiblich, n = 294 männlich und n = 25 ohne Geschlechtszuordnung, ungewichtet)
(Eine Tatperson)<br>Woher kannten Sie diese Person? War das…<br><br>(Mehrere Tatpersonen)<br>Woher kannten Sie diese Personen? Waren das...	(Eine Tatperson)<br>1: mein(e) Freund(in) bzw. Exfreund(in) in einer festen Beziehung<br>2: ein Freund/Mitschüler/Arbeitskollege oder eine Freundin/Mitschülerin/Arbeitskollegin von mir<br>3: eine neue Bekanntschaft (z. B. in der Disco)<br>4: jemand aus der Familie, Verwandtschaft<br>5: jemand aus der Nachbarschaft<br>6: eine Person, von der ich abhängig war<br>(z. B. eine Betreuungsperson, etwa aus dem schulischen Bereich oder in der Freizeit)<br>7: eine mir unbekannte Person	14-bis 25-Jährige, die sexualisierte Gewalt mit Körperkontakt bejahten (n = 1.440, davon n = 256 bei erster Gewalterfahrung jünger als 14 Jahre und n = 848 bei erster Gewalterfahrung 14 Jahre oder älter, ungewichtet)

	9: keine Angabe<br><br>(Mehrere Tatpersonen, Mehrfachnennungen möglich)<br>11: mein(e) Freund(in) bzw. Exfreund(in) in einer festen Beziehung<br>12: Freunde/-innen, Mitschüler/-innen, Arbeitskollege/-innen von mir<br>13: neue Bekanntschaften (z. B. in der Disco)<br>14: jemand aus der Familie, Verwandtschaft<br>15: jemand aus der Nachbarschaft<br>16: Personen, von denen ich abhängig war (z. B. Betreuungspersonen, etwa aus dem schulischen Bereich oder in der Freizeit)<br>17: mir unbekannte Personen<br><br>99: keine Angabe	
**Anwesenheit eines oder mehrerer Bystander**		
Haben Sie schon einmal selbst mitbekommen, dass eine andere Person zu sexuellen Handlungen gedrängt oder gezwungen wurde?	Mehrfachnennungen möglich<br><br>11: nein, sowas habe ich nicht mitbekommen<br>12: ja, ich habe es selbst gesehen/war in der Situation dabei<br>13: ja, die betroffene Person hat es mir erzählt<br>14: ja, die Tatperson hat es mir erzählt<br>15: ja, andere Personen, die davon mitbekommen haben, haben es mir erzählt<br><br>99: keine Angabe	Alle Befragten<br>(n = 5.855, davon n = 3.556 weiblich, n = 2.256 männlich und n = 43 ohne Geschlechtszuordnung bzw.<br><br>n = 418 mind. vorwiegend homosexuell/bisexuell orientiert und n = 5.313 mind. vorwiegend heterosexuell orientiert, ungewichtet)
Waren andere/weitere Personen außer den Tatpersonen in der konkreten Situation in der Nähe und haben etwas davon mitbekommen?	Mehrfachnennungen<br><br>11: nein<br>12: ja, Erwachsene<br>13: ja, Jugendliche<br>14: ja, Kinder<br><br>99: keine Angabe	14-bis 25-Jährige, die sexualisierte Gewalt mit Körperkontakt bejahten (n = 1.440, davon n = 256 bei erster Gewalterfahrung jünger als 14 Jahre und n = 848 bei erster Gewalterfahrung 14 Jahre oder älter, ungewichtet)

Bundesinstitut für Öffentliche Gesundheit

**Disclosure-Prozess**		
Haben Sie jemandem von diesem Erlebnis erzählt?	1: ja, direkt danach<br>2: ja, aber erst einige Tage später<br>3: ja, aber erst einige Wochen/Monate später<br>4: ja, aber erst nach Jahren<br>5: nein, niemandem<br><br>9: keine Angabe	14-bis 25-Jährige, die sexualisierte Gewalt mit Körperkontakt bejahten (n = 1.440, ungewichtet)
Was hat Sie veranlasst, mit niemandem zu sprechen?<br><br>*(angelehnt an die SPEAK-Studie, vgl. Maschke/Stecher 2018*)*	Mehrfachnennungen möglich<br><br>11: Ich habe mich geschämt.<br>12: Ich hatte Angst vor Rache.<br>13: Ich wusste nicht, mit wem.<br>14: Ich hatte zu niemandem Vertrauen.<br>15: Mir hätte sowieso niemand geglaubt.<br>16: Ich wollte selbst nicht mehr daran denken.<br>17: Ich dachte, dass ich es damit noch schlimmer machen würde.<br>18: Ich hatte Angst, dass die anderen mir aus dem Weg gehen würden.<br>19: Ich fand es nicht so schlimm.<br>20: Es gab andere Gründe: Welche? Bitte kurz beschreiben.<br><br>98: Ich weiß es nicht<br>99: keine Angabe	14-bis 25-Jährige, die Offenlegung über Erlebnis sexualisierter Gewalt mit Körperkontakt verneinten (n = 324, davon n = 134 männlich, n = 187 weiblich und n = 3 ohne Geschlechtszuordnung, ungewichtet)
Wem haben Sie davon erzählt?	Mehrfachnennungen möglich<br><br>11: Mutter oder Vater<br>12: Lehrer/Lehrerin<br>13: Therapeuten/Therapeutinnen, Sozialarbeiter/ -arbeiterinnen oder Personen aus Fachberatungsstellen<br>14: Leiter/Leiterin einer Jugend- oder Sportgruppe<br>15: Ärzten/Ärztinnen<br>16: Polizei<br>17: einem anderen Erwachsenen<br>18: dem (damaligen) Partner/Freund bzw. Partnerin/Freundin<br>19: jemandem aus meinem Freundeskreis<br>98: jemand anderem: Wem? Bitte angeben<br><br>99: keine Angabe	14-bis 25-Jährige, die Offenlegung über Erlebnis sexualisierter Gewalt mit Körperkontakt bejahten (n = 1.039, davon n = 688, bei denen Sexualität zu Hause thematisiert wird und n = 320, bei denen Sexualität zu Hause nicht thematisiert wird bzw.<br><br>n = 537, bei denen Verhütung zu Hause thematisiert wird und n = 494, bei denen

		Verhütung zu Hause nicht thematisiert wird bzw.<br><br>n = 190 bei erster Gewalterfahrung jünger als 14 Jahre und n = 700 bei erster Gewalterfahrung 14 Jahre oder älter, ungewichtet)
Alles in allem gesehen – hat Ihnen das Gespräch/haben Ihnen diese Gespräche geholfen?<br><br>*(angelehnt an die SPEAK-Studie, vgl. Maschke/Stecher 2018*)*	11: nein, gar nicht geholfen<br>12: nein, kaum geholfen<br>13: ja, etwas geholfen<br>14: ja, sehr geholfen<br><br>98: hätte gerne noch mit jemand anderem gesprochen/mehr Hilfe erhalten: Bitte angeben<br><br>99: keine Angabe	14-bis 25-Jährige, die Offenlegung über Erlebnis sexualisierter Gewalt mit Körperkontakt bejahten (n = 1.039, davon n = 190 bei erster Gewalterfahrung jünger als 14 Jahre und n = 700 bei erster Gewalterfahrung 14 Jahre oder älter, ungewichtet)

*Maschke S, Stecher L (2018) Sexuelle Gewalt: Erfahrungen Jugendlicher heute. Beltz, Weinheim/Basel.
